# Lymph node ratio as a predictor for outcome in oral squamous cell carcinoma: a multicenter population-based cohort study

**DOI:** 10.1007/s00784-020-03471-6

**Published:** 2020-08-04

**Authors:** Steffen Spoerl, Michael Gerken, Andreas Mamilos, René Fischer, Stefanie Wolf, Felix Nieberle, Christoph Klingelhöffer, Johannes K. Meier, Silvia Spoerl, Tobias Ettl, Torsten E. Reichert, Gerrit Spanier

**Affiliations:** 1 Department of Cranio-Maxillofacial Surgery, University Hospital Regensburg, D-93042 Regensburg, Germany; 2 Tumor Center—Institute for Quality Management and Health Services Research, University of Regensburg, Regensburg, Germany; 3 Institute of Pathology, University Regensburg, Regensburg, Germany; 4 Department of Otorhinolaryngology, University Hospital Regensburg, Regensburg, Germany; 5 Department of Otorhinolaryngology, St. Elisabeth Hospital Straubing, Straubing, Germany; 6 Regensburg Center for Interventional Immunology, University Hospital Regensburg, Regensburg, Germany; 7 Department of Internal Medicine 5-Hematology/Oncology, Friedrich-Alexander University Erlangen-Nürnberg, Erlangen, Germany

**Keywords:** Oral squamous cell carcinoma, Lymph node ratio, Lymph node density, Survival, Recurrence

## Abstract

**Objectives:**

Recently, multiple studies addressed the importance of lymph node ratio (LNR) in specifying patients’ risk of disease recurrence in various malignancies. The present study examines the prognostic significance of LNR in predicting outcome of oral squamous cell carcinoma (OSCC) patients after surgical treatment with curative intent.

**Methods:**

Here, we describe a retrospective population-based cohort with 717 patients previously diagnosed with OSCC. Histopathologically verified lymph node metastasis was diagnosed in 290 patients. Among these patients, we evaluated the impact of LNR on overall survival (OAS) and recurrence-free survival (RFS) in uni- as well as multivariate analysis.

**Results:**

A median cutoff (0.055) in LNR was found to significantly predict outcome in OSCC patients. Five-year OAS was 54.1% in patients with a low LNR, whereas a high LNR was associated with a 5-year OAS of 33.3% (*p* < 0.001). Similar results were detected for RFS with a 5-year survival rate of 49.8% (LNR low) and 30.3% (LNR high) (*p* = 0.002). Results were confirmed in multivariate Cox regression which substantiated the importance of LNR in predicting survival in OSCC patients.

**Conclusions:**

LNR was shown to be an independent prognostic factor for outcome of OSCC in a population-based cohort in uni- as well as multivariate analysis. Hereby, a LNR ≥ 0.055 predicted a shorter OAS and RFS in our cohort.

**Clinical relevance:**

Besides established histopathological factors, LNR can be used as a reliable predictor of outcome in OSCC and might therefore be further applied in evaluating adjuvant treatment after resection in curative intention.

## Introduction

Oral squamous cell carcinoma (OSCC) is one of the most common tumor entities worldwide.

With over 200,000 newly diagnosed cases, it accounts for nearly one-third of all head and neck cancers [1]. Therapeutic approaches are primarily based on combination of surgery, radiotherapy, and chemotherapy, depending on site and stage [2]. Apart from the HPV status, biomarkers to predict the outcome of OSCC patients, particularly in advanced stage disease, are still of utmost importance [3, 4]. Especially in patients with advanced disease, adjuvant treatment remains essential [5], including novel therapeutic approaches particularly in metastatic and recurrent disease: Immunotherapy thereby provides promising treatment options for OSCC patients and consecutively got implemented in routine patient care over the last years [6]. Only recently, the KEYNOTE-048 phase 3 study focused on Pembrolizumab in HNSCC patients with metastatic or recurrent disease. Hereby, Pembrolizumab was characterized as an appropriate first-line therapy for patients with recurrent or metastatic HNSCC [7].

However, immunotherapeutic approaches are, from today’s point of view, no adequate adjuvant treatment options in comparison with radiotherapy or radiochemotherapy. With these traditional adjuvant treatment modalities remaining essential, therapy-related adverse events should carefully taken into account. Relating thereto, the positive prognostic effect of adjuvant treatment needs to be further evaluated and confirmed for different individual subgroups of OSCC.

To determine cervical lymph node metastasis, the tumor-node-metastasis (TNM) staging system contains several parameters including size, localization, and, to a certain extent, number of affected lymph nodes [8, 9]. With up to 40% of OSCC patients presenting lymph node metastasis, a thoroughly performed neck dissection remains an essential aspect in treatment of OSCC [10]. However, the extent of each neck dissection is currently not taken into account when using the TNM classification system, which is still crucial for therapy evaluation and planning treatment strategies [11]. However, alternative staging systems were recently taken into account when predicting outcome in OSCC, especially regarding lymph node ratio (LNR) or the number of positive lymph nodes as potential prognostic factors [12–14].

LNR combines information of dissected nodes as well as the number of positive lymph nodes after histopathological examination. It is defined by the ratio of positive lymph nodes and the entire number of dissected lymph nodes. In various tumor entities, LNR was shown as a valuable prognostic parameter by potently predicting patients’ outcome [15–17].

Even though single center studies already revealed the impact of LNR as a prognostic factor in predicting outcome of OSCC patients after resection with curative intent [12, 18, 19], we conducted a multicenter cohort study with a population-based approach in Eastern Bavaria to further evaluate the prognostic impact of LNR in OSCC.

## Materials and methods

### Patient selection

A multicenter retrospective cohort study was conducted utilizing the database of the Tumor Center Regensburg for the region of Eastern Bavaria. This region of Germany, including the districts of Upper Palatinate and Lower Bavaria, represents a population of around 2.3 million people. The study involved only adult individuals residing in the aforementioned region. All patients had been examined and treated for a newly diagnosed OSCC between 2004 and 2017. Diagnostic workup and consecutive treatment were evaluated at three different medical centers: the Department of Cranio-Maxillofacial Surgery as well as the Department of Otorhinolaryngology, both at the University Hospital Regensburg, and the Department of Otorhinolaryngology at the St. Elisabeth Hospital Straubing.

Patients with previous neck dissection or primary radio(chemo)therapy of head and neck squamous cell carcinoma were excluded. A total of 717 patients were included in the study. These patients did not undergo neoadjuvant treatment and underwent surgical resection of the primary lesion to negative margins. The mean resection margin was 5 mm for patients with detailed information from the histopathological report. Additionally, every patient received a neck dissection with a varying extent of included cervical levels, based on the primary tumor site and preoperative radiological findings. Staging was performed according to the UICC guidelines in the 7th edition [20]. Patient data was retrieved from medical records, including age, gender, positive smoking and alcohol anamnesis, tumor site, tumor-node-metastasis (TNM) stage, grading, and surgical and adjuvant therapy. Age-adjusted Charlson comorbidity index (ACCI) was calculated as previously described and without taking OSCC into account [21].

Adjuvant treatment was based on the recommendation of the multidisciplinary tumor board, and radiotherapy or radiochemotherapy was used accordingly. Hereby, frequent indications for adjuvant treatment were given by higher pT-stages, perinodal invasion, or a pN+ status. However, some patients were not eligible for an adjuvant radiochemotherapeutical approach due to impaired medical conditions. Thence, a sole adjuvant radiotherapy was applied. Some patients refused to adjuvant treatment anyway.

Disease relapse was defined as locoregional disease recurrence or distant metastasis by radiologic evidence with clinical correlation or histopathological confirmation by biopsy. Data concerning recurrence-free survival (RFS) and overall survival (OAS) were obtained from medical records, death certificates, registration offices, and the Clinical Cancer Registry of the Tumor Center—Institute for Quality Management and Health Services Research, University of Regensburg, Germany. According to the approval of this study by the institutional ethics committee, patient data was collected completely anonymized. The end of observation was defined as June 30, 2019. Median follow-up time for the entire cohort was 89 months.

### Statistics

LNR was defined by the ratio of positive lymph nodes and the entire number of dissected lymph nodes and was used as a categorized variable. For outcome analysis, median and quartiles were used as cutoff values for categorizing LNR. Metric variables were analyzed for differences in their means using student’s *t* test in case of normal distribution, otherwise using Mann-Whitney *U* test. Independence of categorical variables was analyzed using Pearson’s chi-square test. Overall and recurrence-free survival time was calculated from date of resection to date of death, date of first recurrence, or date last alive until the end point. Survival analyses were performed using the Kaplan-Meier and Cox regression methods. Differences in outcome estimates were tested using the log-rank test. For risk adjustment, multivariate Cox regression was applied, adjusting for demographical, clinical, and histopathological variables, pT- and pN-status, grading, age, gender, and alcohol and nicotine abuse. Results were reported with hazard ratios (HRs) and 95% confidence intervals (CIs). A *p* value < 0.05 was considered significant for all tests. All analyses were performed using IBM SPSS Statistics Version 25.0 (IBM Corp., Armonk, N.Y., USA).

## Results

In total, 717 OSCC patients were included in this retrospective analysis, and the clinicopathological features are summarized in Table 1. Of patients, 71.8% were male, and mean age was 60.8 years (median 60.1, range 28–91 years). Most patients had a positive history of nicotine (75.0%) and alcohol abuse (68.6%), and most tumors were located at the floor of the mouth and tongue (68.4%).

**Table 1:** Clinicopathological characteristics in complete cohort (*n* = 717), UICC 7th edition

		*N*	%
Sex	Female	202	28.2
	Male	515	71.8
Age at diagnosis	< 50	103	14.4
	50.0–59.9	253	35.3
	60.0–69.9	217	30.3
	70.0–79.9	114	15.9
	> 80	30	4.2
Smoking	No	179	25.0
	Yes	538	75.0
Alcohol	No	225	31.4
	Yes	492	68.6
Age-adjusted Charlson comorbidity index	0	74	10.3
	1	148	20.6
	2	155	21.6
	3	116	16.2
	4	83	11.6
	5	59	8.2
	6+	82	11.4
Anatomical site	Buccal mucosa	51	7.1
	Upper alveolus and gingiva	22	3.1
	Lower alveolus and gingiva	106	14.8
	Hard palate	48	6.7
	Tongue	210	29.3
	Floor of mouth	280	39.1
Tumor size	T1	290	40.4
	T2	236	32.9
	T3	56	7.8
	T4	135	18.8
Cervical lymph node metastasis	N0	427	59.5
	N1	110	15.3
	N2a	8	1.1
	N2b	113	15.8
	N2c	50	7.0
	N3	9	1.3
Grading	G1	51	7.1
	G2	528	73.6
	G3/4	138	19.2
UICC stage	I	219	30.5
	II	117	16.3
	III	115	16.0
	IV	266	37.1
Adjuvant therapy	No	382	53.3
	Radiotherapy	232	32.4
	Radiochemotherapy	103	14.4
Death	Alive	395	55.1
	Dead	322	44.9
Death or recurrence	Alive without recurrence	353	49.2
	Death or recurrence	364	50.8
	Total	717	100.0

In 290 patients (40.4%), lymph node metastasis was diagnosed after tumor resection, and most tumors were staged UICC class IV (37.1%). Of patients, 46.8% received adjuvant therapy, with only 103 patients (14.4%) receiving adjuvant radiochemotherapy (Table 1).

In patients with histopathological verification of lymph node metastasis (*n* = 290), neck dissection resulted in a median lymph node yield of 38.0 (range: 1–104). The median of positive lymph nodes was 2 (range: 1–41), resulting in a median LNR of 0.055 (range: 0.011–1.00) (Table 2). Differences in clinicopathological characteristics in patients with positive lymph nodes according to bivariate LNR distribution were evaluated: Here, significant differences were seen for ACCI (*p* = 0.048), extent of neck dissection (*p* = 0.002), pT- and pN-stage (*p* = 0.004, *p* < 0.001), UICC stage (*p* < 0.001) as well as histopathological verification of extranodal spread (*p* < 0.001) (Table 3).

**Table 2:** Characteristics of dissected lymph nodes in patients with cervical lymph node metastasis (*n* = 290)

	Mean	Median	SD	Minimum	Maximum
Lymph node yield	39.6	38.0	19.2	1	104
Number of positive lymph nodes	3.1	2.0	4.8	1	41
Lymph node ratio	0.09044	0.05481	0.11061	0.01111	1.00000

**Table 3:** Patients clinicopathological characteristics according to lymph node ratio (*n* = 290)

		Lymph node ratio						
		Low		High		Total		*χ*^2^
		*N*	%	*N*	%	*N*	%	*ρ*
Sex	Female	37	12.8	39	13.4	76	26.2	0.789
	Male	108	37.2	106	36.6	214	73.8	
Age at diagnosis	< 50	28	19.3	21	14.5	49	16.9	0.325
	50.0–59.9	52	35.9	48	33.1	100	34.5	
	60.0–69.9	39	26.9	39	26.9	78	26.9	
	70.0–79.9	23	15.9	28	19.3	51	17.6	
	> 80	3	2.1	9	6.2	12	4.1	
Smoking anamnesis	No	29	10.0	33	11.4	62	21.4	0.567
	Yes	116	40.0	112	38.6	228	78.6	
Alcohol anamnesis	No	36	12.4	44	15.2	80	27.6	0.293
	Yes	109	37.6	101	34.8	210	72.4	
Age-adjusted Charlson comorbidity index	0	20	6.9	12	4.1	32	11.0	0.048
	1	36	12.4	24	8.3	60	20.7	
	2	29	10.0	27	9.3	56	19.3	
	3	24	8.3	29	10.0	53	18.3	
	4	16	5.5	13	4.5	29	10.0	
	5	10	3.4	15	5.2	25	8.6	
	6+	10	3.4	25	8.6	35	12.1	
Anatomical site	Buccal mucosa	5	1.7	16	5.5	21	7.2	0.069
	Upper alveolus and gingiva	5	1.7	5	1.7	10	3.4	
	Lower alveolus and gingiva	21	7.2	16	5.5	37	12.8	
	Hard palate	10	3.4	14	4.8	24	8.3	
	Tongue	39	13.4	46	15.9	85	29.3	
	Floor of mouth	65	22.4	48	16.6	113	39.0	
Neck dissection side	Left	10	3.4	25	8.6	35	12.1	0.002
	Right	13	4.5	24	8.3	37	12.8	
	Bilateral	122	42.1	96	33.1	218	75.2	
Tumor size	T1	45	15.5	25	8.6	70	24.1	0.004
	T2	61	21.0	58	20.0	119	41.0	
	T3	10	3.4	25	8.6	35	12.1	
	T4	29	10.0	37	12.8	66	22.8	
Cervical lymph node metastasis	N1	93	64.1	17	11.7	110	37.9	< 0.001
	N2a	6	4.1	2	1.4	8	2.8	
	N2b	33	22.8	80	55.2	113	39.0	
	N2c	11	7.6	39	26.9	50	17.2	
	N3	2	1.4	7	4.8	9	3.1	
Grading	G1	5	1.7	2	0.7	7	2.4	0.184
	G2	112	38.6	104	35.9	216	74.5	
	G3/4	28	9.7	39	13.4	67	23.1	
UICC stage	I	0	0.0	1	0.3	1	0.3	< 0.001
	II	0	0.0	0	0.0	0	0.0	
	III	78	26.9	16	5.5	94	32.4	
	IV	67	23.1	128	44.1	195	67.2	
Adjuvant therapy	No	36	12.4	27	9.3	63	21.7	0.326
	Radiotherapy	71	24.5	71	24.5	142	49.0	
	Radiochemotherapy	38	13.1	47	16.2	85	29.3	
Extranodal spread	No	123	84.8	89	61.4	212	73.1	< 0.001
	Yes	22	15.2	56	38.6	78	26.9	
	Total	145	100.0	145	100.0	290	100.0	
Death	Alive	67	23.1	49	16.9	116	40.0	0.031
	Dead	78	26.9	96	33.1	174	60.0	
Death or recurrence	Alive without recurrence	62	21.4	42	14.5	104	35.9	0.014
	Death or recurrence	83	28.6	103	35.5	186	64.1	
	Total	145	50.0	145	50.0	290	100.0	

Definition for low (LNR ≤ median) and high (LNR > median), UICC 7th edition

**Fig. 1:**
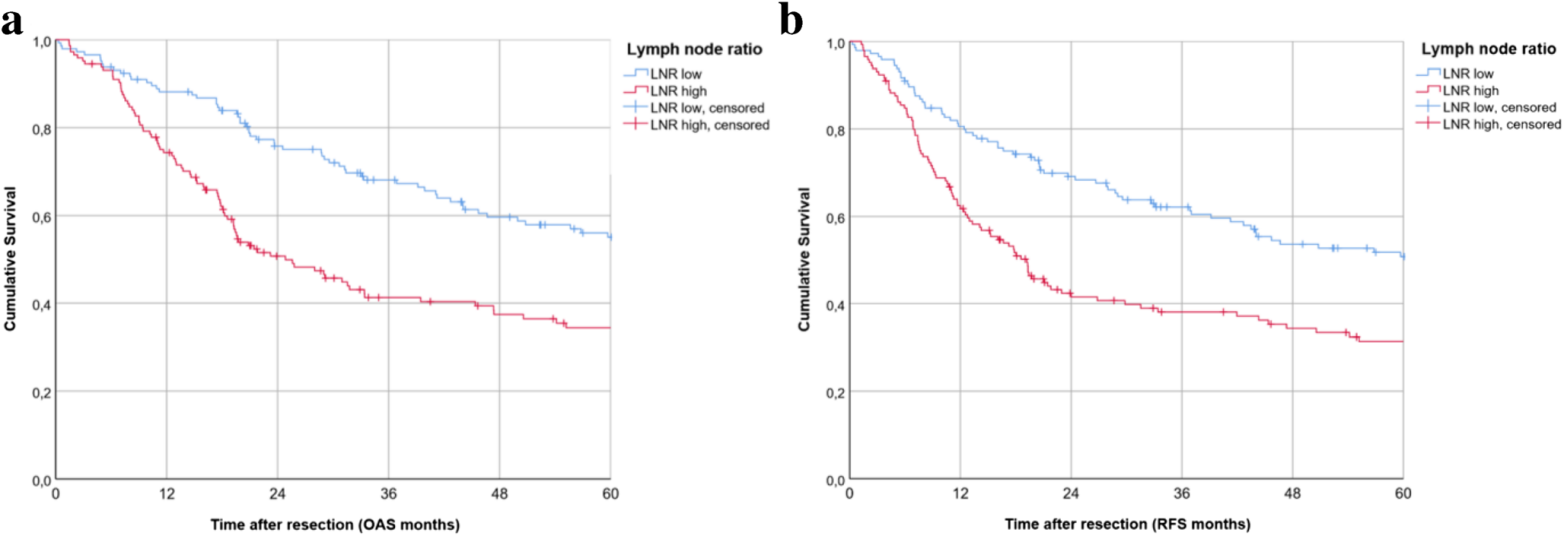
Survival in OSCC patients: Kaplan-Meier curves for OAS (*p* = 0.001) (**a**) and RFS (*p* = 0.002) (**b**) for LNR low (≤ 0.055) and high (> 0.055)

Kaplan-Meier survival analysis in patients with lymph node metastasis revealed a 5-year OAS of 54.1% for LNR ≤ 0.055. In contrast, a LNR > 0.055 resulted in a 5-year OAS of 33.3% in our retrospective cohort (*p* = 0.001). For RFS in patients with lymph node metastasis, survival analysis displayed a 5-year RFS, using a median cutoff in LNR, of 49.8% for a LNR ≤ 0.055. A LNR > 0.055 resulted in a 5-year RFS of 30.3% (*p* = 0.002). Kaplan-Meier curves are shown in Fig. 1 a and b. Results of impaired survival in case of a LNR > 0.055 were substantiated by multivariate Cox regression. Hereby, a LNR > 0.055 resulted in a significantly restricted OAS (HR = 1.492; 95% CI 1.026–2.168, *p* = 0.036) and RFS (HR = 1.503; 95%CI 1.049–2.155, *p* = 0.027) in our retrospective cohort (tables not shown).

Additionally, we not only differentiated LNR by a median cutoff. For further survival analysis, a categorization according to quartiles was applied (LNR < 0.03, LNR = 0.03–0.055, LNR = 0.055–0.107, LNR > 0.107). Survival analysis revealed differences in 5-year OAS for the lowest 25% (53.5% 5-year OAS), the 25–50% (52.7%), the 50–75% (39.7%), and the highest 75% (25.1%) in LNR distribution.

**Fig. 2:**
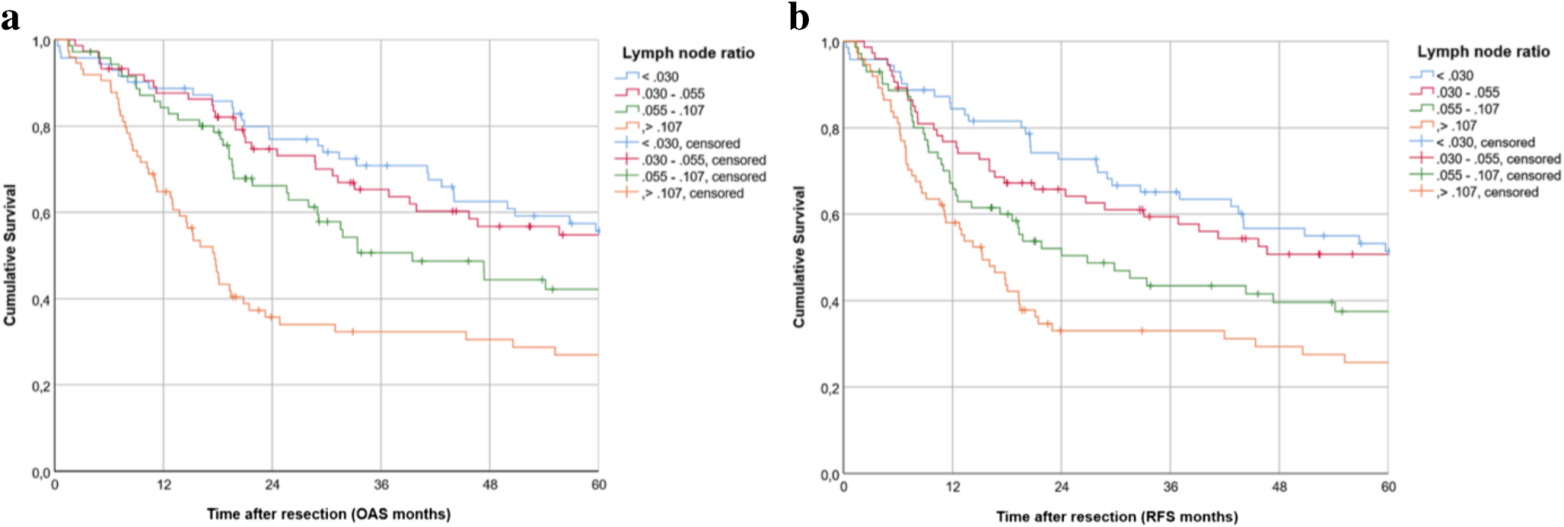
Survival in OSCC patients: Kaplan-Meier curves for OAS (**a**) and RFS (b) for LNR with quartile cutoff points

Similar results were seen for 5-year RFS for the lowest 25% (49.2% 5-year RFS), the 25–50% (48.6%), the 50–75% (35.2%), and the highest 75% (23.8%) (Fig. 2 a and b).

Beyond univariate survival analysis, we performed multivariate Cox regression analysis for quartiles in LNR distribution with risk adjustment for pT- as well as pN-status, grading, age, gender, alcohol as well as nicotine abuse, adjuvant therapy, and extranodal spread. Table 4 shows results of uni- as well as multivariate Cox regression for OAS depending on LNR classified in quartiles in the subcohort of patients with lymph node metastasis. Hereby, univariate Cox regression displayed significant differences in OAS for pT- and pN-stage, age and extranodal spread with significantly impaired (decreased) survival times for more advanced pT-stages (pT3: *p* = 0.021, pT4: *p* = 0.009), a pN3-status (*p* = 0.001), advanced patients age (*p* = 0.039), and histopathological verification of extranodal spread (*p* < 0.001). After risk adjustment by multivariate Cox regression, a high pTN-stage could not be linked to a shorter OAS unlike results of univariate analysis. However, an elevated LNR (LNR > 0.107) hereby clearly impaired OAS of patients with lymph node metastasis when compared with a lower ratio (HR = 1.921; 95% CI 1.080–3.419, *p* = 0.026) (Table 4). Both univariate and multivariate analyses show a clear trend of HRs towards a lower OAS with increasing LNR (Table 4).

**Table 4:** Uni- and multivariate Cox regression for LNR and prognostic factors on OAS (*n* = 290), UICC 7th edition

Variable	Category	Univariate Cox regression				Multivariate Cox regression			
		*ρ*	HR	Lower	Upper	*ρ*	HR	Lower	Upper
				95% CI	95% CI			95% CI	95% CI
LNR (quartiles)	< 0.030		1.000				1.000		
	0.030–0.055	0.854	1.043	0.667	1.631	0.752	1.088	0.645	1.837
	0.055–0.107	0.274	1.280	0.822	1.991	0.658	1.140	0.638	2.035
	> 0.107	< 0.001	2.137	1.413	3.231	0.026	1.921	1.080	3.419
Tumor size	T1		1.000				1.000		
	T2	0.069	1.461	0.971	2.198	0.239	1.292	0.843	1.979
	T3	0.021	1.870	1.100	3.180	0.247	1.428	0.781	2.611
	T4	0.009	1.820	1.161	2.851	0.019	1.784	1.102	2.889
Cervical lymph node metastasis	N1		1.000				1.000		
	N2	0.145	1.266	0.922	1.738	0.700	0.908	0.556	1.483
	N3	0.001	3.851	1.730	8.575	0.645	1.275	0.453	3.587
Grading	G1/2		1.000				1.000		
	G3/4	0.214	1.247	0.880	1.767	0.406	1.167	0.810	1.681
Age at diagnose	< 50		1.000				1.000		
	50.0–59.9	0.383	0.814	0.512	1.293	0.151	0.699	0.429	1.139
	60.0–69.9	0.287	1.278	0.813	2.010	0.527	1.172	0.716	1.917
	70.0–79.9	0.004	2.035	1.254	3.300	0.055	1.678	0.988	2.850
	> 80	0.039	2.207	1.042	4.678	0.697	1.205	0.472	3.078
Sex	Female		1.000				1.000		
	Male	0.122	0.770	0.553	1.072	0.038	0.646	0.428	0.975
Alcohol anamnesis	No		1.000				1.000		
	Yes	0.807	0.958	0.682	1.347	0.412	1.200	0.776	1.857
Smoking anamnesis	No		1.000				1.000		
	Yes	0.680	0.926	0.641	1.337	0.424	1.201	0.766	1.884
Adjuvant therapy	No		1.000				1.000		
	Radiotherapy	0.551	0.891	0.608	1.304	0.306	0.807	0.535	1.217
	Radiochemotherapy	0.181	0.744	0.481	1.148	0.055	0.611	0.369	1.011
Extranodal spread	No		1.000				1.000		
	Yes	0.000	1.821	1.313	2.527	0.008	1.713	1.150	2.552

## Discussion

For patients diagnosed with OSCC, receiving surgical resection of the primary tumor, negative margins and a cervical lymph node dissection represent state-of-the-art therapy [2]. However, lymph node metastasis has been reported as one of the most important prognostic factors in OSCC [22]. Therefore, the pN-status is an essential aspect of current staging systems [8], differentiating between numbers of affected lymph nodes, dimension of lymph node metastasis, and site of occurrence [9]. Unfortunately, the pN-status has several limitations due to different surgical techniques and discrepancies in the extent of neck dissection and histopathological examination of dissected nodes. Various authors therefore postulate that a neck dissection should entail a defined number of dissected lymph nodes. While the 8th edition of the American Joint Committee on Cancer (AJCC) staging manual regards a lymph node yield of at least 15 nodes as an adequate extent for a neck dissection [23], some authors recommend a nodal yield of at least 18 lymph nodes in an elective neck dissection to obtain a beneficial prognosis for OSCC patients [24].

In our retrospective cohort including 717 patients with primarily resected OSCC, lymph node metastasis occurred in 290 patients (40.4%). Hereby, neck dissection resulted in a median lymph node yield of 38 lymph nodes, which certainly represents, in comparison with previously published results [12], an adequate number.

Nevertheless, the number of histopathologically positive lymph nodes among the total amount of dissected nodes was already proposed as a predictive marker for outcome in head and neck cancer in 1994 [25]. Recently, numerous authors characterized the number of positive lymph nodes as an independent prognostic factor for OSCC [13, 26]. Even though the occurrence of positive lymph nodes is taken into account in the current TNM classification for OSCC [9], no further differentiation is made if more than one lymph node is affected.

In this regard, an alternative lymph node staging system is of utmost importance. LNR might ensure both information: the number of lymph nodes being dissected and those being positive for lymph node metastasis. The beneficial aspect of LNR takes the differential extent of each individual neck dissection into account. It results in a simple ratio which can be calculated based on each individual lymph node number*.* Hereby, a less extensive neck dissection is reported to entail a higher LNR, which might impair patients’ outcome [18].

Over the last years, numerous authors examined the potential prognostic value of LNR on various subsites of head and neck cancer [27, 28]. To date, there is presently growing evidence in OSCC that LNR might outmatch conventional nodal staging systems [3, 18, 29]. In this regard, our univariate survival analysis shows that a pN3-status was significantly correlated with decreased OAS in a retrospective cohort analysis. However, this effect is not entirely confirmed in multivariate analysis (Table 4). For LNR, uni- as well as multivariate analysis revealed a significant correlation of higher LNRs with impaired patients’ survival in OSCC (Table 4). In multivariate analysis, LNR might therefore surpass the conventional pN-classification.

In the present study, we investigate LNR as a potential predictor of survival in OSCC patients with lymph node metastasis. A cutoff in LNR was either used as the median distribution or a quartile cutoff was used for both univariate and multivariate survival analysis. Especially the median cutoff point of 0.055 in LNR is similar to the results being reported by different groups. Hereby, a LNR cutoff point of 0.06 was shown to significantly differentiate survival outcome in OSCC [18, 30].

Limitations of this study are mainly due to its retrospective character. Seven hundred nineteen patients met the inclusion criteria for this analysis. For further evaluation of the outcome within different LNRs, solely patients with histopathological verified lymph node metastasis qualified for our study. Of note, we performed one of the largest multicenter analyses regarding the role of LNR for predicting outcome in OSCC.

However, our analysis might be affected by concealed confounders, such as different treatment standards and surgical methods by the individual treating surgeon. Additionally, histopathological examination of dissected lymph nodes is crucial when it comes to obtaining a reliable basis to determine LNR. Hereby, examination of the surgical specimen was conducted by two experienced, board-certified pathologists. Furthermore, each patient undergoing surgery for OSCC is exclusively treated by a highly trained and experienced attending surgeon, so a comparable high-quality standard of surgical care can be assumed within the presented cohort.

Taken together, we were able to highlight LNR as a prognostic factor, determining outcome of patients with advanced stage OSCC.

## Conclusion

In conclusion, our results indicate the importance of LNR as an independent prognostic parameter for OAS and RFS in OSCC patients with lymph node metastasis. Although the TNM classification already entails a lymph node staging system, the number of dissected lymph nodes and number of lymph node metastasis are currently not taken into account in this traditional staging system. However, we were able to clearly demonstrate that LNR is a valuable prognostic parameter in a population-based multicenter cohort study of OSCC patients in Eastern Bavaria. Incorporation of LNR as a prognostic marker in future staging systems might help to stratify patients’ risk and in fact facilitates decision about the need of adjuvant treatment modalities.
